# Expedited diagnosis of pediatric tuberculosis using Truenat MTB-Rif Dx and GeneXpert MTB/RIF

**DOI:** 10.1038/s41598-023-32810-2

**Published:** 2023-04-28

**Authors:** Urvashi B. Singh, Manjula Singh, Sangeeta Sharma, Neeraj Mahajan, Kiran Bala, Abhinav Srivastav, Kh Jitenkumar Singh, M. V. V. Rao, Rakesh Lodha, Sushil K. Kabra

**Affiliations:** 1 Department of Microbiology, All India Institute of Medical Sciences, New Delhi, India; 2 Division of Epidemiology and Communicable Diseases, Indian Council of Medical Research, New Delhi, India; 3 Department of Pediatrics, National Institute of TB and Respiratory Diseases, New Delhi, India; 4 National Institute of Medical Statistics, ICMR, New Delhi, India; 5 Department of Pediatrics, All India Institute of Medical Sciences, New Delhi, India

**Keywords:** Clinical microbiology, Molecular biology

## Abstract

Rapid, cost-effective, and sensitive diagnostic assays are essential for global tuberculosis (TB) control, especially in high TB burden, resource-limited settings. The current study was designed to evaluate diagnostic accuracy of Truenat MTB-Rif Dx (MolBio) in children less than 18 years of age, with symptoms suggestive of TB. Gastric aspirate, induced sputum, and broncho-alveolar lavage samples were subjected simultaneously to AFB-smear, GeneXpert MTB/RIF, liquid culture (MGIT-960) and Truenat MTB-Rif Dx. The index-test results were evaluated against microbiological reference standards (MRS). Truenat MTB-Rif Dx had a sensitivity of 57.1%, specificity of 92% against MRS. The sensitivity and specificity of the Truenat MTB-RIF Dx compared with liquid culture was 58.7% and 87.5% while GeneXpert MTB/RIF was 56% and 91.4%. The performance of both GeneXpert MTB/RIF and Truenat MTB-Rif Dx are comparable. Result of our study demonstrates that Truenat MTB-Rif can aid in early and efficient diagnosis of TB in children.

## Introduction

Pulmonary and intra-thoracic tuberculosis (TB) in children is difficult to diagnose due to the pauci-bacillary nature of disease and varied presentation of involvement of the lungs. More often, younger children present with progressive primary disease (PPD, lung parenchymal lesion) while older children have mediastinal involvement and primary pulmonary complex (PPC, hilar-lymphadenopathy) on Chest X-ray^1^. Issues such as poor/inadequate sample and lower diagnostic yield of laboratory methods further leads to confusion in diagnosis and hence overuse of anti-tubercular treatment for presumptive TB cases.

Detection of bacterial DNA is simpler than detecting live bacteria, which are present only in difficult to reach pulmonary cavities or lymph nodes, with active replication. Recent advancements in molecular diagnostic methods such as Nucleic acid amplification tests (NAATs), e.g. GeneXpert has brought a sea change in the diagnosis of pediatric intra-thoracic TB (ITTB). However, GeneXpert requires a highly sophisticated laboratory with ambient temperature between 15 and 30 °C, stable electricity supply, and adequate storage space for the cartridges (storage at 2–28 °C), thereby, limiting its use to district hospital settings. Certain newer NAATs have shown promise in studies done in older patients^2^. MolBio Diagnostics (Goa, India) developed three different assays namely Truenat MTB, Truenat MTB Plus and Truenat MTB-RIF Dx based on chip-based real-time Polymerase Chain Reaction (PCR) that utilizes *nrdB* gene (Truenat MTB), *nrdz* and *IS6110* gene (Truenat MTB Plus) for the semi-quantitative detection of Mycobacterium tuberculosis (MTB) and *rpoB* gene (Truenat MTB-RIF Dx) for the detection of rifampicin resistance. The Truenat MTB-RIF Dx assay is a novel point-of-care test, operated on hand held device (battery operated), with minimal operational requirements and potential to be utilized in resource limited settings. The assay involves automated extraction of DNA using bead-based Trueprep device that utilizes a universal cartridge-based system to isolate DNA in 20 min, consumables storage 2–40 °C. The DNA is then loaded onto the chip-based Truelab micro PCR device and PCR results concluded in 40 min. In the absence of optimal diagnostic methods, microbiological and clinical composite reference standards are often helpful in diagnosis of TB in children as well as in extrapulmonary TB. In the present study, consensus case definitions were used to ensure standardized reporting of cases of ITTB for research studies^3^. Updated definitions now include GeneXpert MTB/Rif assay as a diagnostic tool for defining Confirmed TB, in addition to culture as part of microbiological confirmation. Current study was designed to evaluate the diagnostic performance of Truenat and GeneXpert in comparison to liquid culture in smear negative and positive specimens and the yield of Truenat MTB-RIF Dx assay in comparison to the microbiological reference standards, GeneXpert MTB/RIF assay, MGIT 960 culture and smear, recommended for accurate diagnosis of ITTB in children^3^.

## Materials and methods

This multicenter, prospective, cohort study was conducted from August 2018 to August 2019. The study population comprised children less than 18 years of age attending Pediatrics OPD at 2 centers in north India. Children less than 18 years age, presenting with symptoms suggestive of TB, such as cough, fever, loss of weight, were screened for TB by clinical examination, X-ray Chest and enrolled after obtaining written, informed consent from parents or guardian. Gastric aspirate (GA), induced sputum (IS), bronchoalveolar lavage (BAL) samples were collected and subjected simultaneously to AFB smear, GeneXpert MTB/RIF, Truenat MTB-RIF Dx and liquid culture by MGIT-960 (LC). Two samples collected on consecutive days from each patient were pooled and subjected to testing. All conventional procedures for smear, LC and GeneXpert MTB/RIF were performed following standard Recommendations^4–6^.

## Specimen processing

Specimens were processed by N-acetyl-L-cysteine sodium hydroxide (NALC-NaOH) method. The clinical samples were treated with NALC, 4% sodium hydroxide and sodium citrate for 15 min followed by neutralization with phosphate-buffered saline (PBS) and centrifugation at 3000 rpm for 30 min. The supernatant was then discarded and pellet was re-suspended in 2 ml of PBS.

### AFB smear

Both direct and decontaminated samples were used for smear preparation. The smears were stained by Ziehl–Neelsen technique and examined under light microscope.

### Liquid culture

The decontaminated samples were inoculated into MGIT 960 (Becton Dickinson, MD, USA). The tubes were incubated at 37 °C. The tubes flagged as positive were tested for contamination on Mueller Hilton Agar (MHA) plate and were confirmed by smear microscopy and TBc identification test (Becton Dickinson, MD, USA).

### Liquid culture drug susceptibility testing (DST)

Drug susceptibility testing (DST) for rifampicin (RIF) and isoniazid (INH) was performed with the MGIT 960 system, using WHO recommended standard critical concentration of 1 µg/ml RIF and 0.1 µg/ml INH. Standard protocol was followed according to the manufacturer's instructions.

### GeneXpert MTB/RIF

The GeneXpert assay was performed according to manufacturer's instructions (Cepheid, Sunnyvale, CA). An aliquot of the digested sample (1 to 2 ml) was frozen at −80 °C at the time of culture processing. After thawing, the specimen was re-suspended by vortex mixing, and 1 ml volume was used according to the instructions of the manufacturer. Briefly, the diluted sample was mixed with 2 ml of GeneXpert sample reagent, inverted 10 times, and incubated for 15 min at room temperature; inversion was repeated after the first 8 min. This mixture was then transferred into the cartridge and loaded onto the GeneXpert instrument. The GeneXpert Dx software (version 4.0, Cepheid) reports results as Mycobacterium complex (MTBC) detected, not detected, invalid, error or no result. The resistance to RIF was reported as RIF resistance detected, not detected or indeterminate. The indeterminate result was reported when the test could not accurately determine whether the bacteria were resistant to RIF. The test was repeated for invalid, error, no result or indeterminate results for RIF resistance.

### Truenat MTB and Truenat MTB/RIF

Truenat MTB and Truenat MTB/RIF were performed as per the manufacturer's instructions (MolBio Diagnostics, Goa, India). The samples were decontaminated using the Trueprep AUTO sample pre-treatment pack. Briefly, to 0.5 ml of sample two drops of liquefaction buffer was added. The entire content was transferred to lysis buffer tube and incubated for 5 min. This mixture was then transferred into the cartridge and loaded on to the device. A total of ~ 40 µl DNA was extracted, out of which 6 µl was added to microtube containing freeze dried PCR reagents and allowed to stand for 30–60 s to get a clear solution. A 6 µl of this clear solution was dispensed into the reaction well of the Truenat MTB chip and loaded to the Truenat Uno Dx instrument. The results were displayed as amplification curve on the analyzer screen on a real time basis during the test run. At the end of the test run, a MTB detected, not detected or invalid results were displayed. The test was repeated for invalid result. The Truenat MTB-RIF Dx being a two-step test, Rif chip is tested after initial detection of Mtb^7^. A total of 6 µl of the purified DNA was added to the microtube containing freeze dried PCR reagents and allowed to stand 30–60 s. Out of this 6 µl was dispensed into the reaction well of Truenat MTB/RIF chip and loaded to the Truenat Uno Dx instrument. At the end of the test run, the results were displayed as Rif resistance detected, not detected, indeterminate or error. Indeterminate or error were displayed when the obtained values does not meet the requirements for resistance determination. The test was repeated for sample displaying indeterminate or error.

Samples were coded by the statistician at NIMS, ICMR, New Delhi daily. Technicians tested coded samples on Truenat. All tests were conducted in NABL (National Accreditation Board for Testing and Calibration Laboratories) accredited mycobacteriology laboratory. All experiments were performed in accordance with the National ethical guidelines for biomedical and research health involving human participants, 2017 (available at:https://ethics.ncdirindia.org/ICMR_Ethical_Guidelines.aspx).

Microbiological reference standards (MRS) include LC, smear and GeneXpert MTB/RIF^1,8^. Truenat MTB-RIF Dx and GeneXpert MTB/RIF results were compared to MRS, smear or LC results taken together as well as to liquid culture alone in order to compare their performance.

## Quality control

It was ensured that, there was no deviation from the set protocol. Laboratory ensured high quality of data and documentation for all methods and is accredited by NABL.

### Sample coding and blinding for index test

Two consecutive days’ samples were pooled and sample aliquots were given code numbers, issued daily for respective samples by NIMS expert. Technician running Truenat test were blinded to the identity of the sample and recorded results against the Code number.

### Data management

Results from all tests were sent back to NIMS. Expert at NIMS was responsible for decoding and final analysis.

### Treatment

The treating physician initiated treatment on the basis of clinical evidence and MRS. Index test results had no bearing on the decision to initiate treatment.

### Statistical analysis

Data were analysed using Stataver 14.1 (SataCorp, College Station, TX). Performance of Truenat MTB-RIF Dx was determined statistically in comparison with GeneXpert MTB/RIF, LC and smear using STATA. Data analysis was done per patient. Further both the molecular tests were compared with gold standard culture (with and without smear results).

### Ethics

Institute Ethics Committee (IEC), All India Institute of Medical Sciences, New Delhi approved the study protocol (IEC No. IEC- 15/2017). TB treatment decisions were not made based on the result of the Truenat MTB-RIF Dx assay under evaluation, but on the basis of the clinical work-up along with MRS.

## Results

### Participant demographics

A total of 612 patients were enrolled during the study period. Out of these, 300 were male while 312 were female patients. The mean age of the patients enrolled was 9.7 ± 3.5 years. Further, the mean age of patients positive by AFB smear was 11.9 ± 2.8, 11.1 ± 2.9 by LC, 11.2 ± 3.4 by GeneXpert MTB/RIF, and 10.9 ± 3.2 years by Truenat MTB-RIF Dx.

**Figure 1:**
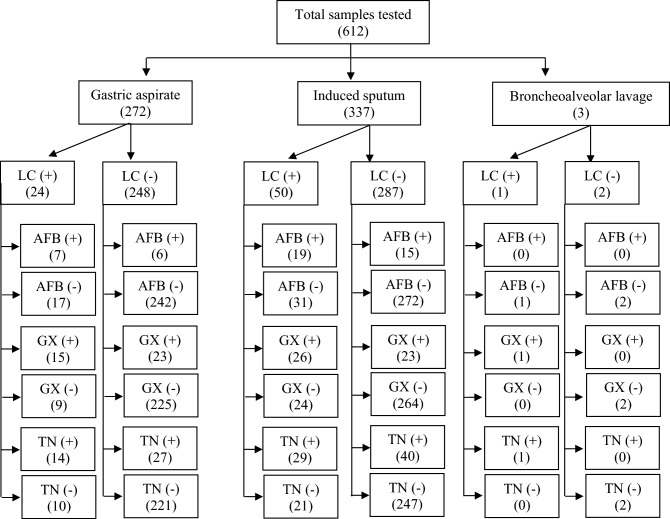
Results of liquid culture (LC), AFB smear (AFB), GeneXpert (GX) and Truenat tests for all samples.

From these children, 272 pooled gastric aspirates (GA), 337 pooled induced sputum samples (IS), and 3 bronchoalveolar lavage (BAL) samples were studied. AFB smear, LC, GeneXpert MTB/RIF and Truenat MTB were performed in all the samples collected. Culture was positive in a total of 75 (12.25%) samples, 24 in GA, 50 in IS and 1 in BAL. AFB was positive in 47 (7.68%) while GeneXpert MTB/RIF and Truenat MTB were positive in 88 (14.38%) and 111 (18.14%) specimens respectively. GeneXpert detected 46 extra positive in comparison to culture, of which 26 were positive by Truenat MTB while Truenat MTB detected 67 extra positive in comparison to culture, of which 26 were positive by GeneXpert MTB/RIF (Fig. 1). Furthermore, Genexpert MTB/RIF was negative in 33 culture positive samples,14 of these were positive by Truenat MTB while Truenat MTB was negative in 31 culture positive samples, 12 of these were positive by GeneXpert MTB/RIF.

### Diagnostic accuracy of truenat MTB Detection assays

The sensitivity and specificity of Truenat MTB was 58.7% and 87.5% while that of GeneXpert MTB/RIF was 56% and 91.4% when compared with MGIT culture (Table 1). The Truenat demonstrated higher sensitivity of 53.1% however somewhat lower specificity of 89.5% in AFB negative specimens while GeneXpert MTB/RIF had a sensitivity of 36.7% and specificity of 94.2%. Additionally, the sensitivity and specificity of Truenat MTB in AFB positive specimens was 69.2% and 39.1% while that of GeneXpert was 92.3% and 23.8% (Table 2). The two molecular tests against smear or LC taken together were 59.4%, 89.5% for Truenat MTB-RIF Dx and 60.4%, 94.2% for GeneXpert MTB/RIF (Table 3).

**Table 1:** Diagnostic performance of Truenat & GeneXpert with MGIT culture.

Truenat	MGIT			GeneXpert	MGIT		
	Pos	Neg	Total		Pos	Neg	Total
Pos	44	67	111	Pos	42	46	88
Neg	31	470	501	Neg	33	491	524
	75	537	612		75	537	612
Sensitivity	58.7 (46.7, 69.9)			Sensitivity	56 (44.1, 67.5)		
Specificity	87.5 (84.4, 90.2)			Specificity	91.4 (88.7, 93.7)		
Positive predictive value	39.6 (30.5, 49.4)			Positive predictive value	47.7 (37, 58.6)		
Negative predictive value	93.8 (91.3, 95.8)			Negative predictive value	93.7 (91.3, 95.6)		
Positive likelihood ratio	4.7 (3.51, 6.31)			Positive likelihood ratio	6.54 (4.65, 9.2)		
Negative likelihood ratio	0.47 (0.36, 0.62)			Negative likelihood ratio	0.48 (0.37, 0.62)

**Table 2:** Diagnostic performance of Truenat & GeneXpert in smear positive and negative specimens with MGIT culture.

		MGIT				MGIT		
	Truenat	Pos	Neg	Total	GeneXpert	Pos	Neg	Total
AFB smear (+) (n = 47)	Pos	18	13	31	Pos	24	16	40
	Neg	8	8	16	Neg	2	5	7
		26	21	47		26	21	47
Sensitivity	69.2 (48.2, 85.7)				Sensitivity	92.3 (74.9, 99.1)		
Specificity	39.1 (18.1, 61.6)				Specificity	23.8 (8.22, 47.2)		
Positive predictive value	58.1 (39.1, 75.5)				Positive predictive value	60 (43.3, 75.1)		
Negative predictive value	50 (24.7, 75.3)				Negative predictive value	71.4 (29, 96.3)		
Positive likelihood ratio	1.12 (0.73, 1.71)				Positive likelihood ratio	1.21 (0.93, 1.58)		
Negative likelihood ratio	0.80 (0.36, 1.79)				Negative likelihood ratio	0.32 (0.06, 1.5)		

	Truenat	MGIT			GeneXpert	MGIT		
		Pos	Neg	Total		Pos	Neg	Total
AFB smear (−) (n = 565)	Pos	26	54	80	Pos	18	30	48
	Neg	23	462	485	Neg	31	486	517
		49	516	565		49	516	565
Sensitivity	53.1 (38.3 (67.5)				Sensitivity	36.7 (23.4, 51.7)		
Specificity	89.5 (86.6, 92)				Specificity	94.2 (91.8, 96)		
Positive predictive value	32.5 (22.4, 43.9)				Positive predictive value	37.5 (24, 52.6)		
Negative predictive value	95.3 (93, 97)				Negative predictive value	94 (91.6, 95.9)		
Positive likelihood ratio	5.07 (3.52, 7.3)				Positive likelihood ratio	6.32 (3.81, 10.5)		
Negative likelihood ratio	0.52 (0.38, 0.7)				Negative likelihood ratio	0.67 (0.54, 0.83)

**Table 3:** Diagnostic performance of Truenat & GeneXpert with AFB smear or MGIT culture.

	AFB smear or MGIT	Both AFB smear and MGIT			AFB smear or MGIT	Both AFB smear and MGIT	
Truenat	Pos	Neg	Total	GeneXpert	Pos	Neg	Total
Pos	57	54	111	Pos	58	30	88
Neg	39	462	501	Neg	38	486	524
	96	516	612		96	516	612
Sensitivity	59.4 (48.9, 69.3)			Sensitivity	60.4 (49.9, 70.3)		
Specificity	89.5 (86.6, 92)			Specificity	94.2 (91.8, 96)		
Positive predictive value	51.4 (41.7, 61)			Positive predictive value	65.9 (55, 75.7)		
Negative predictive value	92.2 (89.3, 94.4)			Negative predictive value	92.7 (90.2, 94.8)		
Positive likelihood ratio	5.67 (4.2, 7.67)			Positive likelihood ratio	10.4 (7.08, 15.2)		
Negative likelihood ratio	0.45 (0.36, 0.58)			Negative likelihood ratio	0.42 (0.32, 0.54)		

With MRS, Truenat MTB revealed sensitivity of 57.1% and specificity of 92% (Table 4). The sensitivity, specificity, positive predictive value and negative predictive value of Truenat in comparison with individual components of MRS are shown in Supplementary Table S1.

**Table 4:** Diagnostic performance of Truenat with Microbiological reference standard (MRS).

Truenat	AFB smear orMGITor GeneXpert Pos	AFB smear & MGIT &GeneXpert Neg	Total
Pos	72	39	111
Neg	54	447	501
	126	486	612
Sensitivity	57.1 (48, 65.9)		
Specificity	92 (89.2, 94.2)		
Positive predictive value	64.9 (55.2, 73.7)		
Negative predictive value	89.2 (86.2, 91.8)		
Positive likelihood ratio	7.12 (5.08, 9.97)		
Negative likelihood ratio	0.46 (0.38, 0.57)		

### Diagnostic accuracy of truenat MTB RIF detection assays

When comparing detection of Rif resistance between Truenat MTB-RIF Dx and GeneXpert MTB/RIF, few samples (n = 14) were reported indeterminate by Truenat MTB-RIF Dx (Table 5). Of 14 indeterminate samples, 12 and 10 were negative by LC and GeneXpert MTB/RIF respectively. Amongst the samples tested positive by LC (n = 2), 1 was sensitive while 1 was not tested for RIF resistance. Furthermore, of the samples that were detected positive by GeneXpert MTB/RIF (n = 4), 3 were sensitive while 1 was indeterminate. The indeterminate results for rifampicin detection in our study may be attributed to the low bacterial load in samples. The concordance analysis of phenotypic rifampicin resistance results with Truenat MTB-RIF Dx and GeneXpert MTB/RIF is shown in Table 6.

**Table 5:** Comparative analysis of Truenat RIF test results with GeneXpert RIF.

Truenat RIF	GeneXpert RIF				
	Sensitive	Resistant	Indeterminate	NA*	Total
Sensitive	49	0	0	42	91
Resistant	1	2	0	3	6
Indeterminate	3	0	1	10	14
NA*	30	2	0	469	501
Total	83	4	1	524	612

Concordance: 521/612 = 85.1%.

*Not applicable (Truenat and GeneXpert not detected).

**Table 6:** Comparative analysis of Truenat RIF and GeneXpert RIF with phenotypic rifampicin resistance.

Truenat RIF	RIF			Total	GeneXpert RIF	RIF		Total
	Sensitive	Resistant	Not Done			Sensitive	Resistant	
Sensitive	39	0	0	39	Sensitive	38	1	39
Resistant	2	1	0	3	Resistant	0	3	3
Indeterminate	1	0	1	2				
Total	42	1	1	44	Total	38	4	42

## Discussion

The current study was designed in a large cohort of children, using state of art methods for diagnosis of presumptive TB. Two nucleic acid detection methodologies Truenat MTB- RIF Dx and GeneXpert MTB/RIF demonstrated comparable performance when evaluated against microbiological reference standards. Strengths of the current study include the study design; few studies have looked at rapid diagnostic modalities of TB in children. The current study was designed to offer a quick but reliable solution for diagnosis of children with presumptive TB. Study design ensured inclusion of a spectrum of clinical presentations. The study definitions were derived using standard recommendations^3^. The study used state of art methodologies and WHO recommended NAATs for diagnosis of TB (GeneXpert MTB/RIF), on a large sample size of patients.

MTB culture can detect even low concentrations of organisms. However, the turn-around time for liquid and solid culture systems varies from few days to several weeks. Additionally, liquid culture system requires sophisticated instrumentation, designated laboratory and specifically trained technical staff. This set-up is not feasible for use in remote areas^7^. There is an urgent need for affordable, easy to use, point-of-care molecular diagnostic assays that augment the efforts to treat disease before its spread and irreversible damage to the individual’s health.

The present study is the first prospective, diagnostic comparative analysis of Truenat MTB-Rif Dx in pediatric population for the detection of TB and rifampicin resistance. The study found comparable sensitivity and specificity against microbiological reference standards, thereby confirming its utility as a rapid and accurate assay for diagnosis of presumptive TB in children. The assay results were found equivalent to the GeneXpert MTB/RIF system, in comparison to microbiological reference standards.

The current study demonstrated lower sensitivity but comparable specificity in children as compared to earlier studies in adults, Truenat MTB-RIF Dx demonstrated sensitivity and specificity of 88.3% and 73.8% for detection of MTB in adults^2^. The sensitivity and specificity for detection of Rif resistance in one study was 87.5% and 99.5% respectively^9^. FIND conducted a study in India, Peru, Ethiopia and Papua New Guinea, in peripheral laboratories and primary health centers. Study results confirmed comparable performance of Truenat MTB, MTB Plus and MTB-RIF Dx with culture and GeneXpert MTB/RIF Ultra and GeneXpert MTB/RIF assays at temperatures up to 40 °C and in absence of reliable electricity (with help of battery operated amplification systems)^10^. The pooled sensitivity and specificity of Truenat MTB; Truenat MTB Plus; and Truenat MTB-RIF Dx differed between primary healthcare centre 73% and 97.7%; 79.8% and 96.3%; 84.2% and 94.7% respectively and reference laboratory 79.5% and 97.7%; 83.5% and 95.7%; 84.6% and 96.8% respectively. The performance of Truenat MTB, MTB Plus and GeneXpert MTB/RIF was comparable with sensitivity and specificity of 82% and 97%; 88% and 95% and 86% and 97% respectively. In one of the sites where Ultra testing was performed the sensitivity of Ultra was higher than Truenat MTB and MTB Plus. The sensitivities observed were 72%, 79% and 95% for Truenat MTB, MTB Plus and Ultra with respective specificities were 99%, 98% and 97%.

WHO endorsed the Truenat assays as initial tests to identify TB and detect rifampicin resistance following the FIND study. The assays were recommended at the point-of-care in low resource primary healthcare settings^11^. The cost for Truenat MTB-RIF Dx per sample (eight hundred rupees) is comparable to GeneXpert MTB/RIF (one thousand rupees). This assay when utilized as a point-of-care for TB diagnosis in India, demonstrated improvement in linkage-to-care, increase in life expectancy and cost-effectiveness when compared with AFB smear or GeneXpert MTB/RIF^12^.

Few limitations included, some delays in doing the Rif test, and prolonged storage of extracted DNA leading to possible degradation of DNA as well as low bacterial load in samples^10^ leading to indeterminate results. A recent study reported decline in the percentage of invalid results after short training and assay operations after a median of 10 tests^13^. We were unable to test the discordant results between the two molecular assays using a third molecular assay, though the phenotypic method was used in parallel.

Current study demonstrated comparable performance of Truenat MTB assays to GeneXpert MTB/RIF assay, in children. The study results reiterate the potential utility of this cost-effective test in primary healthcare centers for diagnosis of presumptive TB in children, a difficult to diagnose and treat population. Several studies have demonstrated the use of Xpert MTB/RIF in pediatric population^14–23^. With the results of equivalence and comparable performance shown between the two tests in the current study, the other GeneXpert MTB/RIF study results can be extrapolated. In addition, the manufacturers offer integrated online connectivity, which could facilitate remote monitoring.

## Supplementary Information

Supplementary Information.

## Data Availability

The datasets generated during and/or analysed during the current study are available from the corresponding author on reasonable request.
