# Supplementary material for: Expedited diagnosis of pediatric tuberculosis using Truenat MTB-Rif Dx and GeneXpert MTB/RIF
Source: Sci Rep. 2023 Apr 28;13:6976. doi: 10.1038/s41598-023-32810-2 (PMC10147673; doi:10.1038/s41598-023-32810-2)
Supplement: Supplementary file 1 — Supplementary Information. [file 41598_2023_32810_MOESM1_ESM.docx]

SUPPLEMENTARY TABLE

**Table 1. Comparative analysis of test results with Truenat**

| AFB Smear | | | | |  | MGIT Culture | | | | |  | GeneXpert | | | | |  |
| --- | --- | --- | --- | --- | --- | --- | --- | --- | --- | --- | --- | --- | --- | --- | --- | --- | --- |
| Truenat |  | Pos | Neg | Total | Sensitivity  66 (50.7, 79.1)  Specificity  85.3 (82.1, 88.2)  PPV  27.9 (19.8, 37.2)  NPV  96.7 (94.7, 98.1) | Truenat |  | Pos | Neg | Total | Sensitivity  58.7 (46.7, 69.9)  Specificity  87.5 (84.4, 90.2)  PPV  39.6 (30.5, 49.4)  NPV  93.8 (91.3, 95.8) | Truenat |  | Pos | Neg | Total | Sensitivity  63.6 (52.7, 73.6)  Specificity  89.5 (86.6, 92)  PPV  50.5 (40.8, 60.1)  NPV  93.6 (91.1, 95.6) |
|  | Pos | 31 | 80 | 111 |  |  | Pos | 44 | 67 | 111 |  |  | Pos | 56 | 55 | 111 |  |
|  | Neg | 16 | 485 | 501 |  |  | Neg | 31 | 470 | 501 |  |  | Neg | 32 | 469 | 501 |  |
|  | Total | 47 | 565 | 612 |  |  | Total | 75 | 537 | 612 |  |  | Total | 88 | 524 | 612 |  |
